# Anatomical Pathology, Behavioral, and Physiological Responses Induced by Application of Non-penetrating Captive Bolt Devices in Layer Chickens

**DOI:** 10.3389/fvets.2019.00089

**Published:** 2019-03-28

**Authors:** Rathnayaka Mudiyanselage Amila Subhashinie Bandara, Stephanie Torrey, Patricia V. Turner, Karen Schwean-Lardner, Tina M. Widowski

**Affiliations:** ^1^Department of Animal Biosciences, University of Guelph, Guelph, ON, Canada; ^2^The Campbell Center for the Study of Animal Welfare, University of Guelph, Guelph, ON, Canada; ^3^Department of Livestock Production, Faculty of Agricultural Sciences, Sabaragamuwa University of Sri Lanka, Blihuloya, Sri Lanka; ^4^Department of Pathobiology, University of Guelph, Guelph, ON, Canada; ^5^Department of Animal and Poultry Science, College of Agriculture and Bio Resources, University of Saskatchewan, Saskatoon, SK, Canada

**Keywords:** euthanasia, brain hemorrhage, brain stem reflexes, insensibility, cardiac arrest, brain death, poultry welfare

## Abstract

We evaluated three models of non-penetrating captive bolt devices, Zephyr-E, Zephyr- EXL, and Turkey euthanasia device (TED) for time to loss of sensibility and degree of brain damage during euthanasia in four age groups of male and female layer chickens (10–11, 20–21, 30–35, 60–70 weeks respectively). Latencies to onset of insensibility and cardiac arrest were assessed to detect whether killing birds via these devices was humane and effective. Both gross and microscopic pathology evaluations were conducted to score skull and brain trauma post mortem. All three NPCB devices induced loss of breathing, pupillary reflex and nictitating membrane reflex within 5 s after application in most chickens. Latencies to loss of jaw tone and neck muscle tone were longer in 60–70 weeks old roosters (*p* < 0.05). Younger birds (10–21 week-old) demonstrated the longest time (*p* < 0.0001) to onset of tonic convulsions, time at last movement, cloacal relaxation and cessation of heart beat. A positive correlation (*p* < 0.0001) was found for all three devices between time of cardiac arrest and times to onset of tonic convulsions, last movement, and cloacal relaxation. More than 80% of birds had skin lacerations with external bleeding following application of all 3 devices. Device type did not affect the incidence of skull fractures but higher skull fracture scores were noted in 10–11 week-old birds compared to other ages. Regardless of device type and age, microscopic SDH was most apparent in the brain and proximal spinal cord of all birds. In summary, all three devices caused significant trauma to the midbrain and spinal cord. Results demonstrated that all three devices induce rapid insensibility after application and can be used as a single-step method that results in a humane death in all age groups of layer chickens.

## Introduction

In the poultry industry, there are several reasons for killing birds during production: to prevent suffering from sickness or injury, for disease control, and for stock management. Therefore, on-farm killing is a routine procedure on commercial poultry farms. Animal care guidelines for livestock and poultry require that the methods used for routine killing cause minimal pain and distress (1). Moreover, the killing method should result in rapid and irreversible loss of sensibility (or consciousness) to be considered humane (2).

The most common method for killing poultry on farms is manual cervical dislocation which involves stretching and separating the cervical vertebrae by hand. However, manual cervical dislocation is considered esthetically displeasing to personnel performing it (2), and there is evidence that both manual and mechanical cervical dislocation methods may not cause immediate unconsciousness (3–6). Newly designed euthanasia devices are commercially available and, non-penetrating captive bolt devices (NPCB) have been designed with a blunt bolt head that does not penetrate the brain. A NPCB device is commonly used to stun large mammals such as cattle, slaughter weight pigs and adult sheep, but is not recommended as a sole method of euthanasia for large animals, as it may not cause death as a one-step method of euthanasia and another method is not applied animals may return to sensibility (2, 7).

The efficacy of some NPCB devices has been determined for turkeys. Erasmus et al. (3) compared the prototype Zephyr-E against mechanical cervical dislocation, manual cervical dislocation and blunt force trauma in turkeys. That study demonstrated that the prototype Zephyr-E device and blunt force trauma were more effective in terms of time to loss of sensibility in turkeys compared to manual and mechanical cervical dislocation. These authors further suggested that a NPCB device was more consistent than blunt force trauma at causing insensibility and death in small turkeys. Woolcott et al. (8) studied two commercial models of NPCB devices: Zephyr-EXL and Turkey Euthanasia device (TED) on turkeys at three stages of production and concluded that both devices were highly effective and reliable at inducing immediate insensibility. Gibson et al. (9) evaluated electroencephalographic (EEG) and behavioral responses of turkeys stunned with three different NPCB devices (Cash Poultry Killer, TED, and Zephyr EXL), and concluded that all devices were effective in causing insensibility in turkeys, provided they were positioned correctly with the correct power load.

Insensibility is often assessed using brain stem reflexes including pupillary light and nictitating membrane reflexes in poultry (3, 10, 11). NPCB devices are used to cause damage to the regions of the brain which control consciousness and vital functions. Due to the acceleration force of the physical technique, cerebral contusion results with neuronal damage and internal bleeding. Brain pathological lesions associated with hemorrhage were associated with immediate loss of consciousness and indicative of immediate and irreversible loss of central regulation of breathing and heart function in poultry (4, 8, 12).

There are fewer published scientific evaluations of NPCB devices in layer chickens. Martin et al. (5) evaluated a cartridge-powered NPCB device (Accles and Shelvoke Cash Poultry Killer: Model.22 CPK 200) in layer chickens, and reported 99.1% kill success with the shortest duration to loss of brain stem reflexes compared to manual and mechanical cervical dislocation. However, the Cash Poultry Killer is cartridge-based, heavier, and more difficult to use compared to other NPCB devices, a significant disadvantage. An evaluation of other lighter weight commercially available pneumatically-powered NPCB devices is needed for layer chickens as an alternative for on-farm euthanasia.

The objective of the present study was to compare the efficacy of the Zephyr-E, Zephyr-EXL, and TED, all pneumatically powered NPCB devices, for on-farm euthanasia of four different ages of layer chickens. Latencies to onset of insensibility and cardiac arrest were assessed as to determine whether the techniques were humane and effective. Gross and microscopic evaluation and scoring of the skull and brain were used to assess induced trauma.

Animal care guidelines require that death be confirmed before leaving birds and disposing of carcasses (1, 2). Common criteria include lack of breathing, pulse, and cessation of the heart. Auscultation may be used to monitor heart beat but this can be difficult for stock persons in the field as it requires a stethoscope, skill, and practice. Therefore, identification of behavioral and physiological reflexes that are correlated with cardiac arrest is needed to readily confirm death before carcass disposal. Therefore, this study also investigated practical behavioral and physiological indicators to confirm the death in layer chickens.

## Materials and Methods

The procedures and protocol for this research were reviewed and approved by the University of Guelph Animal Care Committee (AUP 3321), which holds a Good Animal Practice certificate issued by the Canadian Council on Animal Care.

### Animals and Facilities

All chickens enrolled in this study were obtained from different research projects that were conducted at the Arkell Poultry Research Station of the University of Guelph. All the birds had been targeted for euthanasia by researchers or staff, because they had reached end of study or because of routine flock depopulation. The Zephyr-E, Zephyr-EXL, and TED devices were assessed for killing efficacies and humaneness on four age groups of layer chickens: 10–11 weeks (1.1 ± 0.2 kg), 19–20 weeks (1.7 ± 0. 2 kg), 30–35 weeks (1.8 ± 0.2 kg), and 60–70 weeks (2.2 ± 0.2 kg). Approximately 25 birds in each age group were evaluated with each of the three devices, assessing 279 chickens in 4 different strains of White Leghorn, Brown Leghorn, Columbian Rock, and Plymouth Rock (Table 1). Different age groups of birds were available on different days. Birds were randomly assigned to a device, and a random order of application for each device was followed on a trial day. Sex was not balanced among the treatments due to fewer available male layer chickens. All the birds were euthanized at the Arkell Poultry Research Station, University of Guelph.

**Table 1:** List of number of birds killed with the different NPCB devices by age group, strain, body weight, and sex.

**Age (weeks)**	**Device**	**Body wt (kg)**	**Strain**	**Male**	**Female**	**Total**
10–11	Z-E- standard	1.1 ± 0.2	White Leghorn	13	12	25
	Zephyr-EXL	1.1 ± 0.2	White Leghorn	13	12	25
	TED	1.1 ± 0.2	White Leghorn	13	25	25
20–21	Z-E-standard	1.5 ± 0.2	White Leghorn	1	1	2
	Z-E-layer	1.7 ± 0.2	White Leghorn	2	12	
		1.9 ± 0.3	Plymouth Rock	1	2	17
	Zephyr-EXL	1.6 ± 0.1	White Leghorn	3	8	
		1.9 ± 0.3	Plymouth Rock	3	4	18
	TED	1.7 ± 0.2	White Leghorn	3	12	
		2.2 ± 0.9	Brown Leghorn	2	0	
		1.8	Plymouth Rock	0	1	18
30–35	Z-E-standard	1.8 ± 0.2	White Leghorn	0	12+(1)	13
	Z-E-layer	1.9 ± 0.2	Brown Leghorn	0	10+(1)	
		2.0 ± 0.1	Columbian Rock	0	3	14
	Zephyr-EXL	1.7 ± 0.2	White Leghorn	0	12	
		1.9 ± 0.1	Brown Leghorn	0	4	
		1.8 ± 0.1	Columbian Rock	0	9	25
	TED	1.6 ± 0.2	White Leghorn	0	12	
		1.9 ± 0.1	Brown Leghorn	0	6	
		1.8 ± 0.2	Columbian Rock	0	7	25
60–75	Z-E-standard	2.2 ± 0.1	White Leghorn	2+(3)	0	
		2.4 ± 0.4	Brown Leghorn	0	4	
		1.8 ± 0.1	Columbian Rock	0	3	
		2.2 ± 0.1	Plymouth Rock	0	2	14
	Z-E-layer	2.4 ± 0.3	Brown Leghorn	0	8+(2)	
		2.1	Columbian Rock	0	1	
		2.3 ± 0.1	Plymouth Rock	0	5	16
	Zephyr-EXL	2.2 ± 0.1	White Leghorn	14+(1)	0	
		2.4 ± 0.7	Brown Leghorn	0	2	
		2.2 ± 0.2	Columbian Rock	0	7	
		1.9	Plymouth Rock	0	1	25
	TED	2.0 ± 0.1	White Leghorn	14	0	
		2.2 ± 0.2	Brown Leghorn	0	6	
		2.1 ± 0.7	Columbian Rock	0	3	
		2.4 ± 0.1	Plymouth Rock	0	2	25

*Number of failed birds are indicated in parentheses*.

*Z-E-standard, Zephyr-E with standard subject adapter and conical shape bolt head. Z-E-layer, Zephyr-E with chicken subject adapter and round shape bolt head. TED, Turkey euthanasia device*.

### Non-penetrating Captive Bolt Devices

**Figure 1:**
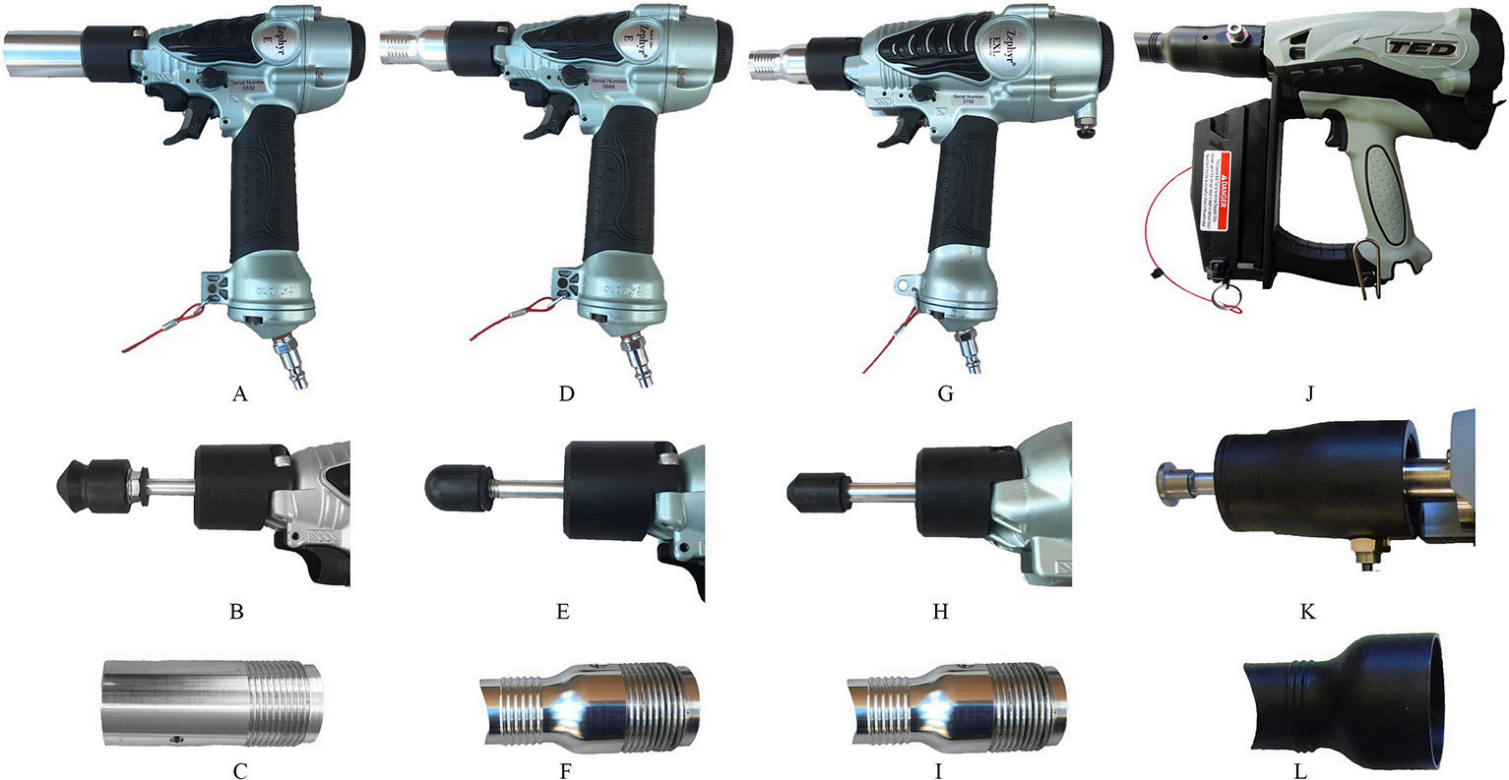
Non-penetrating captive bolt devices. **(A)** Zephyr-E standard: **(B)** Conical shape bolt head, **(C)** Standard subject adapter. **(D)** Zephyr-E-layer: **(E)** Round shape bolt head, **(F)** Chicken subject adapter. **(G)** Zephyr-EXL: **(H)** Conical shape bolt head, **(I)** chicken subject adapter. **(J)** Turkey Euthanasia Device (TED): **(K)** Flat bolt head, **(L)** R-3 subject adapter.

All three NPCB devices were commercially manufactured by Bock Industries, Inc., Philipsburg, PA, USA. The Zephyr-E used in this study is the commercial model weighing 0.75 kg. Two models used of the Zephyr-E device were used in this study: Zephyr-E-standard (Figure 1A) and Zephyr-E-layer (Figure 1D). Zephyr-E consists of a modified pneumatic nail gun fitted with a nylon head (diameter: Zephyr-E standard = 25 mm; Zephyr-E layer = 19 mm) attached to a cylindrical metal bolt (diameter: 9.5 mm). The shape of the bolt head is conical in Zephyr-E-standard (Figure 1B) and round in Zephyr-E-layer (Figure 1E). When fully extended, the bolt protrudes (Zephyr-E-standard = 19 mm; Zephyr-E-layer = 11.3 mm) past the barrel. In both Zephyr-E devices, bolt velocity is 20 m/s, delivering 11 Joules when used at 120 psi (Bock-Industries.com). The Zephyr-E-standard was used with standard subject adapter (Figure 1C), and the Zephyr-E-layer was used with chicken subject adapter (Figure 1F).

The Zephyr-EXL is another commercially available pneumatic-powered NPCB device (Figure 1G). The Zephyr-EXL uses a modified pneumatic nail gun that was fitted with a nylon head (diameter: 25 mm) attached to a conical tipped cylindrical metal bolt (diameter: 9.5 mm) (Figure 1H). The Zephyr-EXL bolt velocity is 27 m/s and delivers 26 Joules when used at 120 psi (Bock-Industries.com). The Zephyr-EXL weighs 0.91 kg. The Zephyr-EXL was used with the chicken subject adapter (Figure 1I).

The Turkey Euthanasia Device (TED) (Figure 1J) is a propane-powered NPCB device. The TED consists of a gas-powered modified nail gun fitted with a flat metal head (diameter: 19.1 mm, length: 4 mm) (Figure 1K) attached to a cylindrical metal bolt (9.5 mm) weighing 1.8 kg (including battery and fuel cell). The TED delivers 28 Joules with a bolt velocity of 30 m/s (Bock-Indusitries.com). The TED was used with adapter R3 (Figure 1L). When the bolt is fully extended, it protrudes 7.1 mm with the R-3 subject adapter.

### Application of the NPCB Devices

**Figure 2:**
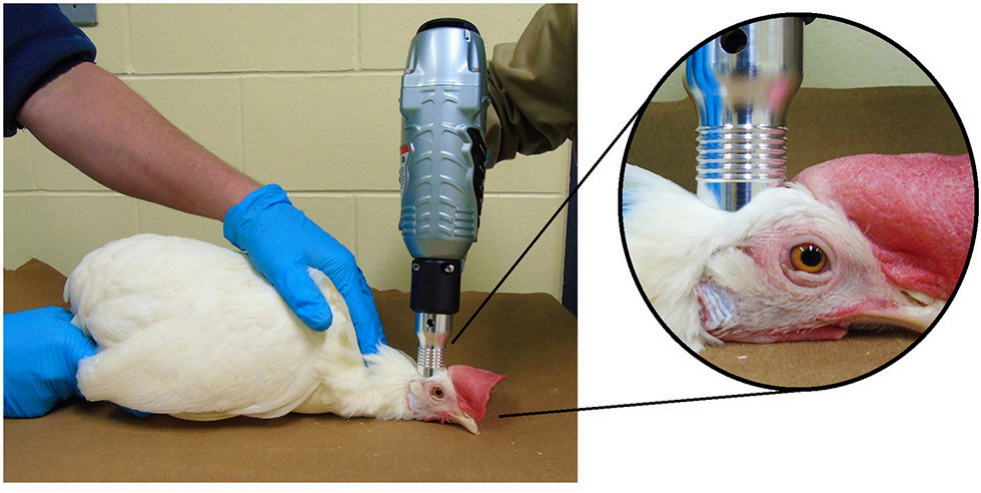
Application of the Zephyr-EXL device on a 30 w.o. hen: The bird was restrained in sternal recumbency with its neck resting ventrally on the ground, and the wings held gently toward the body during the application of the device. Device was placed perpendicular to the top of the frontal bone just behind the comb and on the mid line between the eyes and ears.

Per manufacturer instructions (13), all devices were applied perpendicular to the top of the frontal bone just behind the comb and on a mid-line between the eyes and ears (Figure 2). The Zephyr-E was set to 120 psi by connecting to a compressed air power supply (Hitachi EC 510, Hitachi Koki U.S.A., Ltd: running horse power = 0.8 kW, tank capacity = 22.7 L, maximum pressure = 145 psi). The Zephyr- EXL was set to the 98–100 psi. For consistency, the NPCB device operator was constant throughout all trials. Each bird was positioned in sternal recumbency with its keel on a flat hard surface and restrained by another researcher holding the wings gently toward the body of the bird during the application of the device. Restraint cones were not used in order to facilitate observation of convulsions. A single discharge was administered for each device.

### Ante Mortem Assessments

The measures and procedures used for ante-mortem assessment are presented in Table 2. Time to loss of brain stem reflexes (pupillary reflex and nictitating membrane reflex), jaw tone, and neck muscle tone were assessed to detect insensibility. The parameters were assessed immediately after device application and then at 10 s intervals until cessation. Pupillary reflex was checked for another 30 s (three times) after noting its absence to confirm brain death. If eye reflexes and/or breathing were present more than 60 s following device application, a second discharge was applied immediately, and the euthanasia trial was deemed a failure. Time at onset of tonic convulsions (rigid extension of legs and neck), time at last movement, first feather erection and cloacal relaxation following sporadic opening and closing were also recorded. Presence and duration of heartbeat was monitored through stethoscope, and cardiac arrest was determined to have occurred when no discernible heartbeat could be heard. All procedures to and responses of each bird were video recorded after device application. Times to first feather erection and cessation of all convulsions were based on blinded video recordings. Other measures were collected unblinded by live observations, and the time of each event was reconfirmed using the video recordings.

**Table 2:** Ante-mortem assessment measures, descriptions, and procedures used, listed in order of observation after application of each killing method.

**Measures**	**Description**	**Procedure**
Pupillary light reflex	Constriction of the pupil in response to light	Light from a medical penlight was directed into the eye and pupil constriction was examined
Nictitating membrane reflex	Transient closure of the nictitating membrane in response to mechanical stimulation	The medial canthus of the eye or the cornea was lightly touched with a fingertip
Jaw tone	Resistance to downward pressure applied to the jaw	Gentle pressure was applied to the lower jaw with a finger
Neck muscle tension	Change in neck muscle tone or movement of the head when the neck is lifted	The neck was lifted with the fingers of one hand
Gasping	Paroxysmal opening of the beak	Visual observation for paroxysmal opening of the beak
Feather erection	Sudden erection of feathers, not in response to external stimuli	Visual observation of sudden feather erection on some part of the body
Tonic convulsions	Muscle rigidity with the legs and wings outstretched	Visual observation of the time of onset of legs and neck outstretched
Cloacal relaxation	Cloaca opening following contractions of cloaca	Visual observation for cloaca opening following contractions
Cardiac arrest	Cessation of heart beat	Auscultation by using a stethoscope
Breathing	Rhythmic inhalation and exhalation	Visual observation for rhythmic movement of the chest area

### Macroscopic Assessment of Tissue Damage

**Figure 3:**
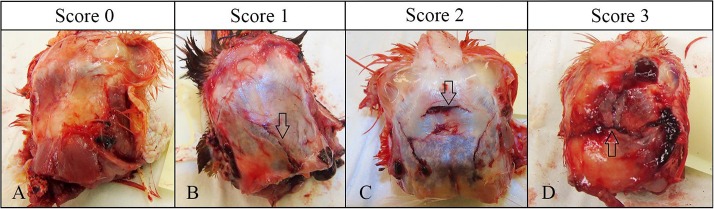
Gross pathology scoring criteria for skull fractures. Arrows indicate the fracture type [modified from Erasmus et al. (12) and Casey-Trott et al. (15)]. **(A)** No fracture, intact skull (score 0). **(B)** Depression fracture (score 1). **(C)** Penetrating fracture-no imbedded fragments (score 2). **(D)** Penetrating fracture- with imbedded fragments (score 3).

Gross pathologic assessment was performed immediately after death in all chickens successfully killed on the first attempt. Degree of external injury caused by each device was assessed (0–2 scale system) based on lacerations of the skin and presence of external hemorrhage at the site of device application: 0 = no laceration of the skin, 1 = laceration of the skin with no external bleeding, 2 = laceration of the skin with external hemorrhage. Presence or absence of bleeding from mouth and nose were also recorded as binary (yes/no) responses. Macroscopic scoring of the degree of skull damage was based on a 0–3 scale system (Figure 3). Subcutaneous hemorrhage (SCH) and subdural hemorrhage (SDH) were assessed based on a 0–4 scale (Table 3) (8, 14). The degree of SCH was assessed by removing the scalp and examining the amount of hemorrhage under the scalp. Then the dorsal surface of the skull was cautiously cleaned, removing blood and tissues to examine the severity of skull damage. Following this, the skull was lifted, and dura were removed to assess the SDH on the brain.

**Table 3:** Gross and microscopic pathology scoring criteria for macroscopic, and microscopic hemorrhage.

**Score**	**Macroscopic**	**Microscopic**
	Subcutaneous or	Subdural or
	subdural hemorrhage	parenchymal hemorrhage
0	None	None
1	<25% of surface area	Minimal (<5% of section)
2	26–50% of surface area	Mild (5–10% of section)
3	51–75% of surface area	Moderate (11–30% of section)
4	76–100% of surface area	Marked (>30% of section)

### Microscopic Assessment of Brain Trauma

Following macroscopic assessment, brains and the cervical spinal cord (from 1st to 3rd cervical bone) were collected from 6 randomly selected birds in each age group per device for microscopic evaluation. Tissues were placed in 10% buffered formalin for at least 14 days before trimming. For consistency, all trimming was performed by one individual. Three sections of the brain (A: cerebrum, B: mid brain and thalamus, C: cerebellum) and the spinal cord (C1: portion under the first cervical bone, C2: portion under the second cervical bone, C3: portion under the third cervical bone) were sampled. Tissue sections were embedded in paraffin, cut 4 μm and stained with hematoxylin and eosin (Animal Health Laboratory, University of Guelph) prior to assessment. Sections were evaluated by a veterinary pathologist blinded to bird age, breed or treatment to determine the degree of SDH and parenchymal hemorrhage (PCH) using a score from 0 to 4: no hemorrhage (0), minimal (<5%) hemorrhage (1), mild (5–10%) hemorrhage (2), moderate (>10–30%) hemorrhage (3), and marked (>30%) hemorrhage (4, 8, 12, 14, 15).

### Statistical Analyses

Statistical analyses were conducted using SAS 9.4 (SAS Institute Inc., Cary, NC, USA). Generalized linear mixed models (GLMM) were used to analyze the fixed effects of device, age, and their interactions on ante mortem evaluations. Least significant means separation was conducted by using the Tukey-Kramer test.

Pearson product-moment correlation coefficients were used to determine the relationships between the different antemortem measures for each device. Regression analysis was conducted to establish the functional relationship between the highly correlated variables by generating the estimates for the intercept and the regression coefficient by using REG procedure of SAS. Heart beat end time was considered as the dependent variable (Y) and the independent variables (X) were time to onset of tonic convulsions, time at cessation of convulsions, and time at cloacal relaxation. The estimated intercept and the regression coefficient for each variable were used to generate the relevant fixed-effect equation to predict the value of heart beat end time for the given level of the independent variable (cloaca contractions, cessation of convulsions and time at onset of tonic convulsions).

Generalized linear mixed models (GLMM) with multinomial distribution and cumulative logit link functions were used to analyze the effect of the device, age, and their interaction on macroscopic and microscopic trauma assessments (multinomial ordinary data). Odds ratios were computed to compare differences in the levels of fixed effects. Data from the three sections of brain from each bird were pooled and the highest score of the three sections of each brain was used for SDH and PCH analyses (8, 14). The same procedure was applied to the SDH and PCH analyses for the spinal cord.

## Results

### Ante Mortem Assessments

All three NPCB devices induced loss of breathing, pupillary reflex and nictitating membrane reflex within 5 s after application in most chickens. Overall, 100% successful killing was observed for the TED with immediate and irreversible insensibility in all age groups. One failure (a 29 w.o. hen) was noted of 93 birds for the Zephyr-EXL for unknown reasons. The Zephyr-E resulted in 7 failures of 101 birds. Two failures occurred in 35 w.o. hens (one with the Zephyr-E-standard and the other with the Zephyr-E-layer) and 5 birds were 65 w.o. (three roosters with the Zephyr-E-standard and two hens with the Zephyr-E-layer) (Table 1). These birds continued to demonstrate a pupillary light reflex, gasping, and rhythmic breathing. After 60 s, the TED was applied as the second killing method. Ante mortem and pathology assessments were not conducted for failed trials.

Clonic convulsions, with severe wing flapping and leg paddling, were observed immediately following device application, regardless of device type, or age group. Feather erection was observed in all successfully killed birds. First feather erection was commonly observed in the neck region, followed by intermittent feather erections in different areas of the body. Time to onset of first feather erection was remarkably consistent between all devices and age groups (Zephyr-E = 41 ± 2 s, Zephyr-EXL = 41 ± 2 s, TED = 43 ± 2 s, *p* = 0.773). Gasping was observed in some successfully killed birds for all three devices (Zephyr-E: 9 out of 94, Zephyr-EXL: 6 out of 91, TED: 3 out of 93). The average duration ± SD duration of gasping for Zephyr-E = 51 ± 28 s, Zephyr-EXL = 29 ± 17 s, and TED = 16 ± 2 s. Two birds killed with the Zephyr-EXL started gasping at 50 s and 90 s after the application of device while others started at 10 s after application of the device. None of these birds demonstrated concurrent pupillary or nictitating eye reflexes.

A summary of ante mortem responses is shown in Table 4. Both time to loss of neck tone and time to loss of jaw tone showed a difference for device, age, and device by age interaction (*p* < 0.05). The longest times to loss of jaw tone (31 ± 3 s) and neck muscle tone (28 ± 3 s) were observed in 60–70 w.o. roosters. The TED had the longest times (*p* < 0.05) to loss of jaw tone (28 ± 3 s) and neck muscle tone (36 ± 6 s). Overall, time to loss of jaw tone and neck muscle tone were longer in 60–70 w.o. roosters for TED (39 ± 6 s and 53 ± 9 s respectively, *p* < 0.05). Younger birds (10–11 week and 20–21 w.o.) showed longest times (*p* < 0.0001) for onset of tonic convulsions and cloacal relaxation (Table 4). A device effect was not observed for times to onset of tonic convulsions or last movement. Onset of tonic convulsions was easily observed (stretched neck and legs with sudden feather erection) in 10–11 w.o. birds compared to the older groups. A longer time to cloacal relaxation (*p* < 0.05) was observed for the TED (186 ± 24 s) compared to the Zephyr-E (167 ± 24 s), and no difference was found between the TED and the Zephyr-EXL. Some birds defecated during cloacal relaxation regardless of the device. As the very last measure, longest time for cessation of heart beat (*p* < 0.0001) was observed in younger birds (10–11 w.o.: 235 ± 26 s). The shortest times for cloacal relaxation (*p* = 0.033) and cessation of heart beat (*p* = 0.035) were recorded for the Zephyr-E.

**Table 4:** Mean time (± SE, s) to onset of specific measures after application of different NPCB devices in different age groups of layer chickens.

**Measure**	**Age (wks)**	**Device**			**All devices**	***p*****-value**		
		**Z-E**	**Z-EXL**	**TED**		**Device**	**Age**	Device^*^Age
Loss of jaw tone						**<0.0001**	**<0.0001**	**0.0063**
	10–11	13 ± 2^bc^	22 ± 2^bc^	25 ± 6^b^	19 ± 3^b^			
	20–21	21 ± 3^bc^	21 ± 2^bc^	29 ± 6^b^	23 ± 3^b^			
	30–35	20 ± 3^bc^	19 ± 2^bc^	24 ± 6^bc^	21 ± 3^b^			
	60–70	28 ± 3^b^	27 ± 2^b^	39 ± 6^a^	31 ± 3^a^			
	All ages	21 ± 3^b^	22 ± 3^b^	28 ± 3^a^				
Loss of neck muscle tone						**<0.0001**	**<0.0001**	**0.0297**
	10–11	18 ± 4^e^	23 ± 5^de^	32 ± 9d^c^	24 ± 6^c^			
	20–21	25 ± 4d^ce^	23 ± 5^de^	33 ± 10^dc^	26 ± 6^b^			
	30–35	27 ± 4d^c^	29 ± 5^dc^	33 ± 10^dc^	30 ± 6^b^			
	60–70	35 ± 4^bc^	44 ± 5^ab^	53 ± 9^a^	46 ± 6^a^			
	All ages	27 ± 6^b^	30 ± 6^b^	36 ± 6^a^				
Time at first feather erection	10–11				38 ± 2	0.7733	0.2154	0.0546
	20–21				45 ± 2			
	30–35				39 ± 2			
	60–70				44 ± 2			
	All ages	41 ± 2	41 ± 2	43 ± 2				
Onset of tonic						0.2909	**<0.0001**	0.8839
	10–11				153 ± 22^a^			
	20–21				141 ± 23^a^			
	30–35				111 ± 23^b^			
	60–70				103 ± 22^b^			
	All ages	122 ± 22	126 ± 22	132 ± 22				
Last movement						0.1042	**<0.0001**	0.9026
	10–11				206 ± 25^a^			
	20–21				189 ± 25^a^			
	30–35				146 ± 25^b^			
	60–70				147 ± 25^b^			
	All ages	164 ± 25	171 ± 25	181 ± 25				
Cloacal relaxation						**0.0335**	**<0.0001**	0.8349
	10–11				203 ± 24^a^			
	20–21				199 ± 25^a^			
	30–35				153 ± 25^b^			
	60–70				150 ± 24^b^			
	All ages	167 ± 24^b^	175 ± 24^ab^	186 ± 24^a^				
Cessation of heart beat						**0.0354**	**<0.0001**	0.8866
	10–11				235 ± 26^a^			
	20–21				209 ± 27^b^			
	30–35				173 ± 27^c^			
	60–70				178 ± 26^c^			
	All ages	189 ± 26^b^	198 ± 26^ab^	209 ± 26^a^				

*Different letters indicate statistically significant (P < 0.05) differences between the comparisons*.

Bold numbers indicate significance results (P < 0.05)

*Z-E = Zephyr-E, Z-EXL = Zephyr-EXL, TED = Turkey euthanasia device*.

The Pearson product-moment correlation coefficient was computed to assess the relationship between the ante mortem measures for each device. Table 5 summarizes the results. Overall, there were strong, positive significant correlations between time to cardiac arrest and time to cloacal relaxation (Zephyr-E: *r* = 0.935, Zephyr-EXL: *r* = 0.906, TED: *r* = 0.906), time to last movement (Zephyr-E: *r* = 0.951, Zephyr-EXL: r = 0.934, TED: *r* = 0.948), and time to onset of tonic convulsions (Zephyr-E: *r* = 0.917, Zephyr-EXL: r = 0.856, TED: r = 0.808). There was a small positive relationship between times to cardiac arrest and loss of neck muscle tone (*r* = 0.34, *p* = 0.0008) and between cardiac arrest and loss of jaw tone (*r* = 0.30, *p* = 0.003) for TED. However, correlations between cardiac arrest vs. loss of jaw tone or loss of neck muscle tone were not significant for the Zephyr-E and Zephyr-EXL devices. Loss of jaw tone and loss of neck muscle tone showed a positive (*p* < 0.0001) correlation for all three devices. Strong positive correlations (*p* = 0.0001) were also found between onset of tonic convulsions and cloacal contraction, onset of tonic convulsions and time to last movement, and cloacal relaxation and time to last movement for all three devices (Table 5). Feather erection was not associated with any other measure for any of the devices evaluated.

**Table 5:** Pearson correlation coefficients to assess the relationship between the antemortem measures for different NPCB devices for all ages of layer females and males (*n* = 94 for Zephyr E, *n* = 92 for Zephyr EXL and *n* = 93 for TED).

**Variable**	**Device**	**Loss of neck muscle tone**	**Loss of jaw tone**	**First feather erection**	**Onset of Tonic convulsions**	**Cloacal relaxation**	**Last movement**
Cessation of heart	Z-E	−0.1082	−0.1056	0.1088	**0.9174**	**0.9352**	**0.9519**
		*p* = 0.2991	*p* = 0.3111	*p* = 0.296	***p*** **< 0.0001**	***p*** **< 0.0001**	***p*** **< 0.0001**
beat	Z-EXL	0.0917	0.0723	0.0703	**0.8566**	**0.9067**	**0.9342**
		*p* = 0.387	*p* = 0.495	*p* = 0.5078	***p*** **< 0.0001**	***p*** **< 0.0001**	***p*** **< 0.0001**
	ZED	**0.3422**	**0.3003**	**0.2065**	**0.8081**	**0.9068**	**0.9482**
		***p*** **= 0.0008**	***p*** **= 0.0034**	***p*** **= 0.047**	***p*** **< 0.0001**	***p*** **< 0.0001**	***p*** **< 0.0001**
Loss of neck muscle tone	Z-E		**0.7948**	−0.0744	−0.2007	−0.1003	−0.1622
			***p*** **< 0.001**	*p* = 0.475	*p* = 0.052	*p* = 0.3383	*p* = 0.1183
	Z-EXL	–	**0.7089**	0.2021	−0.026	0.058	0.0462
			***p*** **< 0.0001**	*p* = 0.054	*p* = 0.8047	*p* = 0.5912	*p* = 0.663
	ZED		**0.7660**	**0.2971**	**0.2371**	**0.2134**	**0.2832**
			***p*** **< 0.0001**	***p*** **= 0.0038**	***p*** **= 0.0221**	***p*** **= 0.0399**	***p*** **= 0.0059**
Loss of jaw tone	Z-E			−0.1461	−0.1467	−0.0639	−0.1299
				*p* = 0.1599	*p* = 0.1583	*p* = 0.5427	*p* = 0.2119
	Z-EXL		–	−0.1128	−0.036	0.0259	−0.0105
				*p* = 0.286	*p* = 0.7289	*p* = 0.8102	*p* = 0.9212
	ZED			0.1880	**0.2371**	**0.2138**	**0.2763**
				*p* = 0.0711	***p*** **= 0.0221**	***p*** **= 0.0396**	***p*** **= 0.0073**
First feather	Z-E				0.1148	0.1903	0.1235
					*p* = 0.2705	*p* = 0.067	*p* = 0.2374
erection	Z-EXL			–	0.0251	0.0487	0.0894
					*p* = 0.8128	*p* = 0.6522	*p* = 0.3989
	ZED				0.0588	0.1013	0.1914
					*p* = 0.5755	*p* = 0.333	*p* = 0.066
Onset of Tonic convulsions	Z-E					**0.9341**	**0.9519**
						***p*** **< 0.0001**	***p*** **= 0.0001**
	Z-EXL				–	**0.9049**	**0.9056**
						***p*** **< 0.0001**	***p*** **< 0.0001**
	ZED					**0.7915**	**0.8195**
						***p*** **< 0.0001**	***p*** **< 0.0001**
Cloacal relaxation	Z-E						**0.960**
							***p*** **< 0.0001**
	Z-EXL					–	**0.94877**
							***p*** **≤ 0.0001**
	ZED						**0.9428**
							***p*** **< 0.0001**

*Z-E, Zephyr-E; Z-EXL, Zephyr-EXL; TED, Turkey euthanasia device*.

*Bolded numbers indicate significant results (P < 0.05)*.

Regression analysis was conducted for the highly correlated variables considering time to cardiac arrest as a dependent variable. Regression equations and relevant coefficient of determinations (R2) are presented in Table 6.

**Table 6:** Regression and relative contribution (R^2^) for response of dependent variable (Y) for independent variables (X) of different NPCB devices.

**Independent variable (X)**	**Dependent variable (Y)**	**Device**	**Regression equation**	**Coefficient of determination (R^**2**^)**
Time at onset	Heart beat	Z-E	Y = 35.77 + 1.1X	0.8416
of tonic convulsions	end time	Z-EXL	Y = 57.15 + 1.1X	0.7339
		TED	Y = 84.52 + 0.9X	0.6532
Time at cloacal	Heart beat	Z-E	Y = 19.1 + 1X	0.8746
relaxation	end time	Z-EXL	Y = 33.6 + 1X	0.8222
		TED	Y = 26.2 + 1X	0.8224
Time at last	Heart beat	Z-E	Y = 24.6 + 1X	0.9062
movement	end time	Z-EXL	Y = 40.5 + 0.9 X	0.8728
		TED	Y = 34.6 + 1X	0.8991

*All regression coefficients were significant (P < 0.05)*.

*Z-E, Zephyr-E; Z-EXL, Zephyr-EXL; TED, Turkey euthanasia device*.

### Pathology Evaluations

#### Macroscopic Evaluation

The degree of external damage (external hemorrhage and skin lacerations) was not different among the devices (*p* = 0.595), age groups (*p* = 0.062), or their interaction (*p* = 0.689). Skin lesions with external bleeding were noted in >80% of birds for all 3 devices (Zephyr-E: 80.6%, Zephyr-EXL: 85.8%, TED:82.8%) and more than 70% in all age groups (10–11 w.o.: 85.3%, 20–21 w.o.: 72.2%, 30–35 w.o.; 81.3%, 60–70 w.o.: 90.4%). No external damage was observed in a few birds killed by the different devices (Zephyr-E: 11.8%, Zephyr-EXL: 4.3%, and TED:12.9%) and in the different age groups (10–11 w.o.: 5.3%, 20–21 w.o.:20.3%, 30–35 w.o.;12 %, 60–65 w.o.:11.1%). Some birds showed both nasal and mouth bleeding (Zephyr-E = 7/94, Zephyr-EXL = 3/92, TED = 0/93) and protruding eyes with the cornea covered with blood (Zephyr-E = 10/94, Zephyr-EXL = 11/92, TED = 18/93).

**Figure 4:**
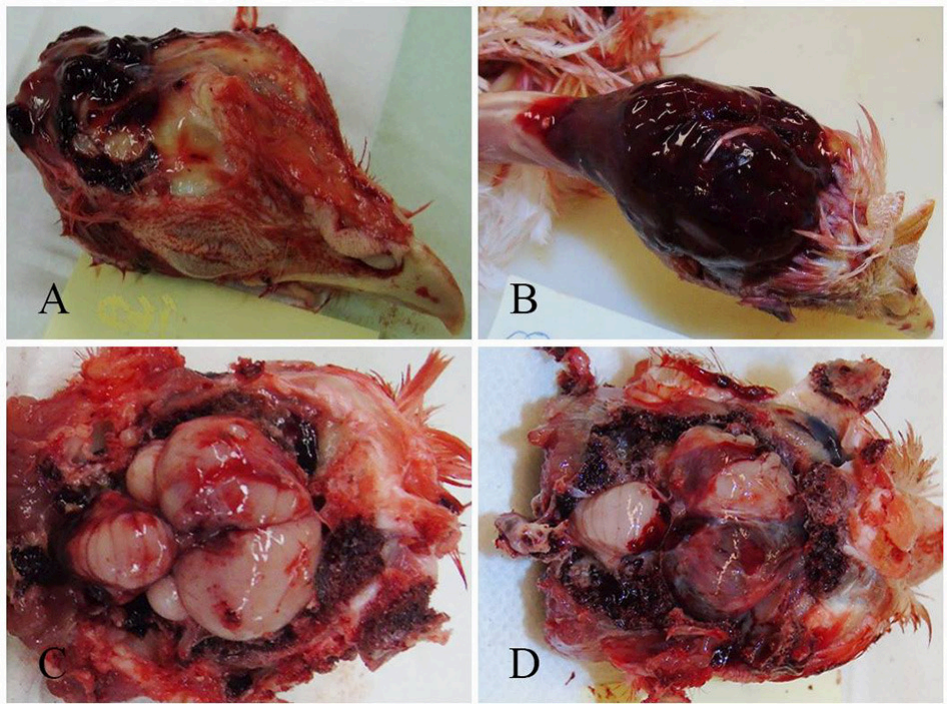
Skin reflected to demonstrate gross subcutaneous hemorrhage. **(A)** Hemorrhage with less than 25% of area covered (score 2) of a 65 w.o. bird killed by the TED. **(B)** Hemorrhage completely covering area from the eyes to base of the skull (score 4) of a 10 w.o. bird killed by the TED. **(C)** Gross subdural dorsal hemorrhage covering <25% of the brain surface (score 1) of a 33 w.o. bird killed by the Zephyr-E. **(D)** Gross subdural dorsal hemorrhage covering 51–75% of the brain surface (score 3) of a 33 w.o. bird killed by the Zephyr-E.

Macroscopic lesion scores are presented in Table 7. All birds had subcutaneous hemorrhage on the skull regardless of the device and age. The highest score of 4 was observed in 99% of birds in the 10–11 w.o. group. Figure 4 shows two skulls with 25 and 100% macroscopic subcutaneous hemorrhage. Macroscopic subcutaneous hemorrhage found on the skull was different among the devices (*p* < 0.0001), age (*p* < 0.0001) and device by age interaction (*p* = 0.029). Lower SCH scores were noted to occur 3.6 times more in birds killed with the Zephyr-E than the TED and 0.1 times more likely for the Zephyr-EXL than the TED. No difference was found between the Zephyr-E and Zephyr-EXL.

**Table 7:** Summary of gross scores for subcutaneous hemorrhage, skull fractures, and subdural hemorrhage in birds killed by different NPCB devices.

	**Device**	**Age**	**Number of birds with gross score**						***p*****-value**		
			**0**	**1**	**2**	**3**	**4**	**Total**	**Device**	**Age**	Device*Age
SCH									**<0.0001**	**<0.0001**	**0.0295**
	Z-E	10–11	0	0	0	2	23	25			
		20–21	0	3	5	4	7	19			
		30–35	0	5	12	6	2	25			
		60–70	0	3	2	10	10	25			
	Z-EXL	10–11	0	0	0	4	21	25			
		20–21	0	2	2	7	7	18			
		30–35	0	2	15	5	3	25			
		60–70	0	7	7	4	6	24			
	TED	10–11	0	0	1	2	22	25			
		20–21	0	0	0	1	17	18			
		30–35	0	2	3	9	11	25			
		60–70	0	1	4	6	14	25			
Skull fractures									0.1485	**0.0001**	**0.0017**
	Z-E	10–11	0	0	20	5	N/A	25			
		20–21	1	4	11	3	N/A	19			
		30–35	0	3	19	3	N/A	25			
		60–70	1	2	13	9	N/A	25			
	Z-EXL	10–11	0	2	14	9	N/A	25			
		20–21	0	2	11	5	N/A	18			
		30–35	0	0	15	10	N/A	25			
		60–70	7	4	5	8	N/A	24			
	TED	10–11	0	0	13	12	N/A	25			
		20–21	0	7	11	0	N/A	18			
		30–35	1	4	13	7	N/A	25			
		60–70	5	5	13	2	N/A	25			
Brain									**<0.0001**	**<0.0001**	**0.0203**
SDH	Z-E	10–11	0	2	13	8	2	25			
		20–21	0	5	7	4	3	19			
		30–35	0	7	10	7	1	25			
		60–70	0	1	12	7	5	25			
	Z-EXL	10–11	0	0	6	10	9	25			
		20–21	0	2	4	5	7	18			
		30–35	0	4	9	10	2	25			
		60–70	0	4	11	7	2	24			
	TED	10–11	0	0	4	6	15	25			
		20–21	0	1	4	10	3	18			
		30–35	0	3	11	9	2	25			
		60–70	0	4	5	7	9	25			

*Number of birds with each score are indicated*.

*Z-E, Zephyr-E; Z-EXL, Zephyr-EXL; TED, Turkey euthanasia device*.

*SDH, Subdural hemorrhage; SCH, subcutaneous hemorrhage*.

*Bolded numbers indicate significant results (P < 0.05)*.

Skull fracture scores differed among the age groups (*p* = 0.0001) and there was an age by device interaction (*p* = 0.0017). Skull fractures were common in the 10–11 w.o. group and more than 98% of birds of this age had penetrating fractures from all devices. Birds in the 20–21 and 60–70 w.o. groups had lower fracture scores than 11 w.o. birds. No difference was found between the 10–11 w.o. vs. 30–35 w.o., and 20–21w.o. birds vs. 60–70 w.o. birds. In addition, there was a 0.4 times greater chance of lower scores in 60–70 w.o. birds than in 30–35 w.o. birds. Some birds in the 60–70 w.o. group did not have any skull fractures (Zephyr-E = 4%, Zephyr- EXL = 29%, TED = 20%). An age difference was found only for TED and not for the Zephyr devices.

Subdural macroscopic hemorrhage on the brain was substantial and 100% of birds had a score of 1 or more for all three devices (Table 7). Over 85% of birds had a score 2 or above for all devices. The highest score of 4 was observed in 12% of birds killed by the Zephyr-E, 21% of birds killed by the Zephyr-EXL and 31% of birds killed by the TED. Figure 4 shows one brain covered by <25%, and another covered by 50–75% of subdural hemorrhage. Subdural macroscopic dorsal hemorrhage was affected by device (*p* < 0.0001), age (*p* < 0.0001), and device x age interaction (*p* = 0.020, Table 7). The Zephyr-E generally resulted in lower scores compared to the TED or Zephyr-EXL. There was no difference in degree of subdural hemorrhage between the TED and Zephyr-EXL. Birds at 10–11 w.o. generally had higher injury scores than all the other age groups. A higher chance of having higher injury scores was observed in birds at 20–21 w.o. than in birds at 30–35 w.o. Birds at 30–35 w.o. had 1.8 times higher chance of having lower trauma scores than birds at 60–70 w.o.

#### Microscopic Evaluation

Table 8 provides a summary for microscopic scores for subdural (SDH) and parenchymal (PH) hemorrhage in three brain sections (cerebrum, mid brain, and cerebellum) killed with different NPCB devices. SDH was observed in the cerebrum of all 24 birds killed by the TED, but only some birds killed by Zephyr-E and Zephyr-EXL. Similarly, PCH was absent in the cerebrum of some birds killed by Zephyr-E (*n* = 4), Zephyr-EXL (*n* = 3) and TED (*n* = 1). SDH was substantial in the midbrain and observed in all assessed birds killed by all three devices. PCH was also found in the mid brains of all birds killed by Zephyr-EXL and TED except one bird killed by Zephyr-E. All birds had SDH in the hind brain except one bird killed by Zephyr-EXL and TED. Nearly 50% of birds did not have PCH in the hind brain when killed by the Zephyr-E (*n* = 11), Zephyr-EXL (*n* = 7) and TED (*n* = 12). PCH was absent in the hind brain of all 6 birds in 10–11 w.o. group. Four of six birds killed by the TED and one of six killed by the Zephyr-EXL in 10–11 w.o. group did not have hind brain PCH. Overall, all three devices caused the highest degree of trauma to the mid brain.

**Table 8:** Summary of microscopic scoring of brains for trauma following application of each of the three NPCB devices in layer chickens.

**Device**	**Brain section**	**SDH Score**					**PCH Score**				
		**0**	**1**	**2**	**3**	**4**	**0**	**1**	**2**	**3**	**4**
Z-E	Cerebrum	5	1	6	6	6	4	6	7	6	1
	Mid brain	0	0	8	5	11	1	9	7	2	5
	Hind brain	0	1	4	17	2	11	6	2	0	5
Z-EXL	Cerebrum	3	0	2	10	9	3	3	4	8	6
	Mid brain	0	2	2	7	13	0	4	4	7	9
	Hind brain	1	4	5	7	7	7	7	7	2	1
TED	Cerebrum	0	0	5	10	9	1	7	6	10	0
	Mid brain	0	2	3	11	8	0	2	5	15	2
	Hind brain	1	1	6	9	8	12	8	4	0	0

*Number of birds with each score are indicated*.

*Z-E, Zephyr-E; Z-EXL, Zephyr-EXL; TED, Turkey euthanasia device*.

*SDH, Subdural hemorrhage; PCH, Parenchymal hemorrhage*.

There were no device, age or their interaction effects on SDH in the brain or spinal cord (Table 9). However, SDH was observed in both the brain and spinal cord for all three devices in all age groups. Similarly, PCH was observed in the brain in all ages for all three devices, but there was a device effect (*p* = 0.024). Birds killed with the Zephyr-EXL had a greater chance of having higher scores for PCH in the brain than those killed with the Zephyr-E or TED. Overall, 46% of birds received a score of 4 for PCH when killed with Zephyr-EXL, compared to 8% for the TED and 29% for the Zephyr-E. There was no difference between the TED and Zephyr-E. In contrast to brain PCH, a device effect was not observed for PCH in the spinal cord (*p* = 0.182). However, PCH in the spinal cord was different (*p* = 0.044) among the age groups and showed an age by device interaction (*p* = 0.021). Birds in the 60 w.o. group had a greater chance of having higher scores for PCH in the spinal cord than birds in the 10–11 or 30–35- w.o. groups.

**Table 9:** Overall summary of microscopic scoring of subdural hemorrhage and parenchymal hemorrhage in the brain and spinal cord of layer chickens killed by NPCB device.

	**Device**	**Age**	**Score**					**Total**	***P*****-Value**		
			**0**	**1**	**2**	**3**	**4**		**Device**	**Age**	Device^*^Age
SDH in brain									1.000	0.5388	0.8972
	Z-E		0	0	0	12	12	24			
	Z-EXL		0	0	1	6	17	24			
	TED		0	0	0	10	14	24			
SDH in spinal cord									0.999	0.995	0.9136
	Z-E		0	0	3	9	12	24			
	Z-EXL		0	1	1	7	15	24			
	TED		0	0	1	7	16	24			
PCH in									**0.0242**	0.1694	0.4794
brain	Z-E		0	7	6	4	07	24			
	Z-EXL		0	1	3	9	11	24			
	TED		0	2	5	15	02	24			
PCH in spinal cord									0.1822	**0.0443**	**0.0215**
	Z-E	10–11	0	3	2	1	0	6			
		20–21	1	3	2	0	0	6			
		30–35	1	1	2	2	0	6			
		60–70	0	5	1	0	0	6			
	Z-EXL	10–11	3	3	0	0	0	6			
		20–21	1	2	1	1	1	6			
		30–35	3	2	1	0	0	6			
		60–70	0	1	4	1	0	6			
	TED	10–11	1	3	1	1	0	6			
		20–21	0	1	4	1	0	6			
		30–35	1	4	1	0	0	6			
		60–70	0	1	3	2	0	6			

*Number of birds with each score are indicated*.

*Z-E, Zephyr-E; Z-EXL, Zephyr-EXL; TED, Turkey euthanasia device*.

*SDH, Subdural hemorrhage; PCH, Parenchymal hemorrhage*.

*Bolded numbers indicate significant results (P < 0.05)*.

## Discussion

To the author's knowledge, this is the first study to evaluate the commercially available pneumatic NPCB devices (Zephyr-E, Zephyr-EXL, TED) for on-farm killing of layer chickens. Our results demonstrated that all three devices were similarly effective at inducing insensibility and causing death in all age groups.

All devices caused loss of pupillary light reflex, nictitating membrane reflex, and breathing within 5 s after application. Direct or indirect (contrecoup) trauma to the brain can result in impairment of brain stem reflexes (16, 17). The area that controls the pupillary light reflex (cranial nerve II and III) is located in the mid brain. The nictitating membrane reflex is controlled by both cranial nerve III (located in the mid brain) and cranial nerve V (located in the pons). It is possible that direct damage to the corresponding cranial nerves caused loss of these reflexes. However, the substantial microscopic parenchymal hemorrhage (PCH) demonstrated that all three devices caused severe trauma to the mid brain. The medulla oblongata in the hind brain contains important regions that regulate the cardiovascular and respiratory systems. The control centers of jaw tone and neck muscle tone are also located in the hind brain. The majority of birds had microscopic SDH (99%) and PCH (more than 50%) in the hind brain. The damage caused by the devices to the hind brain was enough to cause impairment of breathing. Jaw tone and neck muscle tone disappeared in <20 s and 50 s respectively, in all birds, also indicating that all three devices caused hind brain damage. Overall, all three devices disrupted brain function and caused rapid brain death.

Sandercock et al. (10) reported that jaw tone and neck muscle tone were the most reliable reflexes distinguishing between sensible and insensible states in poultry based on EEG studies. Loss of jaw tone has been used as an indicator of loss of sensibility in poultry under field conditions (3, 11). All birds in our study showed jaw tone and neck muscle tone for a few seconds following application of all three devices, and muscle tone did not disappear as quickly as did the eye reflexes. Martin et al. (11) also reported longer time to loss of jaw tone (21.7 s) than pupillary reflex (11.6 s) in chickens killed with a penetrating captive bolt (Modified rabbit zinger®). Results of the present study revealed longer time to loss of jaw tone and neck muscle tone for TED compared to the Zephyr devices, which corresponded to differences in PCH scores in the hind brain between devices. Moreover, the shortest time for cloacal relaxation and cardiac arrest, two responses controlled by the hind brain, were recorded for Zephyr-E. We suggest that the force caused to the hind brain by the TED is different compared to the Zephyr devices. Differences between the TED and Zephyr devices could be due to the shape of the bolt heads which that deliver force differentially across and through the skull; the TED has a flat bolt head and the Zephyr devices have either round or conical bolt head.

Erasmus et al. (3) did not report any gasping in turkeys effectively stunned with the Zephyr-E or blunt force trauma. In contrast, a few successfully killed birds (~10%) showed gasping following application all three devices nearly for 60 s. However, their eye reflexes were completely absent. In the current study, paroxysmal opening of the beak without any chest movement associated with breathing was recorded as gasping. Gasping is not indicative of sensibility and can be present in the absence of auditory evoked potentials (18). Therefore, gasping can be observed both in awake and insensible birds, and while unpleasant to watch, is not necessarily indicative of device failure.

Seven birds failed to lose brain stem reflexes and breathing within 60 s for Zephyr-E. Four of them were killed by the Zephyr-E standard with a common subject adapter and 3 with the Zephyr-E layer with the chicken subject adapter. As the failures with the common subject adapter came first, we considered that the adapter was the cause of the failures. Then the device was switched to Zephyr-E-layer with a chicken subject adapter. The chicken subject adapter allows for a better alignment of the device around the comb of the bird. For the Zephyr-E and Zephyr EXL the bolt velocity is adjustable as a function of air pressure. The pressure in the air compressor was slightly lower than 120 psi when killing these seven birds, and we suspect this could be a cause of the failed euthanasia. Based on this finding, we recommend that the Zephyr-E be used exactly at 120 psi in layer chickens of all ages and weights.

Determining reliable indicators of irreversible brain injury are important for detecting clinical death under field conditions since animal care guidelines require confirmation of death before birds are disposed of (1). We studied times to onset of tonic convulsions, last movement, and cloacal relaxation since these are readily observed indicators that can be used to confirm the irreversible brain injury. Tonic convulsions were observed in all successfully killed birds regardless of the device and thus tonic convulsions can be used to indicate a successful euthanasia. Sudden feather erection was observed first around 60 s post device application and also during the tonic phase in all birds killed successfully. Gerritzen et al. (19) confirmed the death of poultry killed with CO_2_ based on sudden feather erection along with occurrence of tonic convulsions followed by complete muscle relaxation. Heard (20) stated that sudden feather erection during anesthesia of birds is indicative of cardiac arrest. However, in the current study, time at first feather erection was not correlated with cessation of heart beat after killing with two of the three devices, and cannot be considered a reliable indicator of time at cardiac arrest. Similar results were determined by Hernandez et al. (21) who also found that feather erection was not associated with cardiac arrest or isoelectric point using EEG. Cessation of movement has been used to estimate irreversible brain death (3, 19, 22). Time to last movement in the present study was highly correlated with time of cardiac arrest, thereby serving as a reliable on-farm indicator of cardiac arrest. Cloacal relaxation was the last reflex observed before cardiac arrest in all birds, which agrees with Martin et al. (11), and demonstrates its utility as a conservative indicator of death. Cardiac arrest typically occurs after all motion has ceased (22, 23). This coincides with the results in present study as all the birds ceased heart beat after cessation of all movements and reflexes. The presence of a heart beat does not indicate sensibility, and is, itself, a conservative measure. Turner et al. (23) also reported the presence of a heartbeat in poultry several minutes after brain death, as confirmed by use of an EEG. Auscultation of the heart with a stethoscope can be difficult under the field conditions so it can be difficult to be certain of the exact moment of cardiac arrest. Additionally, the heart may continue to beat irregularly for some time (21). Onset of tonic convulsions, last movement, and cloacal contractions can be visually observed in the field. The relationship analysis in this study suggest that cardiac arrest was highly positively correlated with onset of tonic convulsions, time to last movement, and cloacal relaxation for all three NPCB devices and thus, onset of tonic convulsions, last movement, and cloacal relaxation can be used by stock persons to make accurate decisions of successful euthanasia under field conditions.

Younger birds (10–11 w.o. and 20–21 w.o.) had a longer latency to onset of tonic convulsions, last movement, cloacal relaxation, and cardiac arrest. In addition, one, four and six of the six 10–11 weeks old birds killed by the Zephyr-EXL, TED and Zephyr-E, respectively, had no PCH in the hind brain, indicating less trauma to the hind brain. This might explain the longer latencies to onset of tonic convulsions, last movement, cloacal relaxation, and cessation of heart beat in 10–11 w.o. group. The oldest birds in the 60–70 w.o. group who had more mature anatomy (fused and larger size skull, large comb) experienced a longer time to loss of jaw tone and neck muscle tone. Therefore, the placement of the device on the head (the place of the skull where the bolt hit), device configurations and anatomic structure of the head may have affected the degree of damage cause to different regions of the brain.

Overall more than 80% birds showed external damage for all three devices. Some birds had mouth and nose bleeding, and damaged eyes. External bleeding is important for biosecurity measures and esthetic concerns. The fine balance between effectiveness and aesthetics is the key to selection of an appropriate method of euthanasia. Higher external damage caused by the NPCB devices may indicate a need for lower air pressure levels for the Zephyr-EXL based on the age group. However, the Zephyr-E should only be used with the manufacturer-recommended 120 psi to avoid any failures.

Results of the macroscopic pathology assessment indicated that lower subcutaneous and subdural hemorrhage scores were more likely for the Zephyr-E. This may be due to lower force generated by the Zephyr-E than for the Zephyr-EXL and TED. All three devices caused penetrating fractures with no embedded fragments in most birds. Skull fractures are highly associated with severe traumatic brain injuries leading to death in humans (24). Studies in other animal species have reported that skull fractures are often present in animals that were effectively stunned with non-penetrating captive bolt devices (15, 25). However, Erasmus et al. (12) reported that presence or absence of skull fractures did not affect onset of insensibility in turkeys killed by NPCB or blunt trauma. Our results also showed that presence or absence of skull fractures did not influence the effectiveness of inducing insensibility and irreversible brain death. Overall, 15 birds in our study had an intact skull, and all of them showed rapid loss of eye reflexes and irreversible brain death.

There was no difference found for the microscopic assessment of subdural hemorrhage in brain sections among the devices. A device effect was found for PCH in that the Zephyr-EXL (at 98–100 psi) caused higher scores of PCH in the brain than that seen with the TED or Zephyr-E. PCH indicates traumatic brain injuries (TBI). Researchers have suggested that immediate insensibility and irreversible loss of vital functions are associated with subdural and parenchymal hemorrhage in poultry (4, 12). Results from all three devices evaluated in this study confirm this. It is important to note that all three devices also caused SDH and PCH in the cervical area of the spinal cord. Therefore, the force generated by all three devices is sufficient to cause intensive traumatic damage to the brain and to the cervical portion of the spinal cord.

An unexpected problem was encountered with the Zephyr devices for Plymouth Barred Rock chickens. In 2 chickens of this strain, the feathers became stuck between the bolt and the adapter for both Zephyr-E and Zephyr-EXL. Despite this, the birds were both killed effectively with these devices. We suggest that plumage type should be considered when using Zephyr devices with chicken subject adapter.

## Conclusion

This study demonstrated that brain trauma cause by all three NPCB devices, was sufficient to rapidly render the birds insensible, leading to irreversible brain death in all age groups of layer chickens. The Zephyr-E, Zephyr-EXL, and TED devices can be used as a humane single-step euthanasia method for layer chickens. Additionally, we suggest onset of tonic convulsions, last movement, and final cloacal relaxation are good indicators of clinical death in layer chickens in field conditions.

## Data Availability

All datasets generated for this study are included in the manuscript and/or the supplementary files.

## Author Contributions

TW, ST, PT, and KS-L are co-principal investigators and conceived and supervised the study. RB designed the experiment. RB collected the data. PT interpreted all microscopic samples. RB conducted all statistical analysis and wrote the paper. All co-principle investigators contributed to editing and final review.

### Conflict of Interest Statement

TW holds the Egg Farmers of Canada Chair in Poultry Welfare. The remaining authors declare that the research was conducted in the absence of any commercial or financial relationships that could be construed as a potential conflict of interest.
